# Antecedents and Consequences of Misinformation Sharing Behavior among Adults on Social Media during COVID-19

**DOI:** 10.1177/21582440221147022

**Published:** 2023-01-16

**Authors:** Ammara Malik, Faiza Bashir, Khalid Mahmood

**Affiliations:** 1 University of the Punjab, Lahore, Pakistan; 2 Government Graduate College for Women, Township, Lahore, Pakistan

**Keywords:** infodemic, misinformation, disinformation, predictors, gratifications, social media, COVID-19, fake news, WhatsApp, pandemic, rumors, Facebook

## Abstract

Misinformation has been existed for centuries, though emerge as a severe concern in the age of social media, and particularly during COVID-19 global pandemic. As the pandemic approached, a massive influx of mixed quality data appeared on social media, which had adverse effects on society. This study highlights the possible factors contributing to the sharing and spreading misinformation through social media during the crisis. Preferred Reporting Items and Meta-Analysis guidelines were used for systematic review. Anxiety or risk perception associated with COVID-19 was one of the significant motivators for misinformation sharing, followed by entertainment, information seeking, sociability, social tie strength, self-promotion, trust in science, self-efficacy, and altruism. WhatsApp and Facebook were the most used platforms for spreading rumors and misinformation. The results indicated five significant factors associated with COVID-19 misinformation sharing on social media, including socio-demographic characteristics, financial considerations, political affiliation or interest, conspiracy ideation, and religious factors. Misinformation sharing could have profound consequences for individual and society and impeding the efforts of government and health institutions to manage the crisis. This SLR focuses solely on quantitative studies, hence, studies are overlooked from a qualitative standpoint. Furthermore, this study only looked at the predictors of misinformation sharing behavior during COVID-19. It did not look into the factors that could curb the sharing of misinformation on social media platforms as a whole. The study’s findings will help the public, in general, to be cautious about sharing misinformation, and the health care workers, and institutions, in particular, for devising strategies and measures to reduce the flow of misinformation by releasing credible information through concerned official social media accounts. The findings will be valuable for health professionals and government agencies to devise strategies for handling misinformation during public health emergencies.

## Introduction

The COVID-19 pandemic had caused determinant consequences on every aspect of human life. Along with collateral health damages, it gave rise to a global economic recession since World War II. Simultaneously, the unprecedented growth of misinformation labeled “Infodemic” surged on social media. Though existed for centuries, misinformation emerged as a severe concern in the age of social mediaduringCOVID-19 crisis. As the pandemic approached, a massive influx of mixed quality data appeared on social media about its origin, symptoms, transmission pattern, medical interventions, and, more recently, about the Corona vaccine. The statement by WHO’s director-general that, “We’re not just fighting an epidemic; we’re fighting an infodemic” clearly portrayed the worsening situation at that time. For the present study, misinformation is defined as unverified information about COVID-19 shared on social media deliberately or mistakenly (Anspach & Carlson, 2020; Bin Naeem & Kamel Boulos, 2021; Nyhan & Reifler, 2010; Pian et al., 2021; Skafle et al., 2022). It includes misinformation, disinformation, hoaxes, conspiracies, and rumors during the COVID-19 crisis.

Misinformation is an enormous challenge on social media, where the rapid and far-reaching spread of information is just a single click away. Literature has reported practical and negative repercussions of sharing misinformation, which hampered human’s capacity to respond to the crisis. This also created doubts and distrust about social media credibility. Individuals’ skepticism toward information systems during the crisis highlighted the need to investigate the motivating factors of sharing misinformation on social media (Cheng & Chen, 2020; Islam et al., 2020; Malik et al., 2021) and its impact on social, psychological, and physical aspects of human life (Abd-Alrazaq et al., 2020; AlHumaid et al., 2020; Ho et al., 2020). The topic has gained much attention during the global pandemic within the last couple of years.

The COVID-19 pandemic, according to Pian et al. (2021), produced the biggest worldwide economic crisis since World War II and nearly 2.99 million deaths by 2021. Despite the numerous initiatives taken by the WHO and public health organizations to combat the infodemic, including launching campaigns against COVID-19 misinformation, collaborating with social media platforms, and regularly disseminating fact-based information to the general public (such as COVID-19 advice for the public: myth busters), the spread of false information has continued to be rife on a global scale (Pennycook et al., 2020; Tasnim et al., 2020). Despite the fact that misinformation does not require professional verification or review, it has the potential to spread more quickly and farther on social media due to the platforms’ algorithms that favor or highlight popular or desired content. Social media can be used effectively to spread vital health-related information to the global community. This emphasizes the difficult task health authorities face in providing the public with accurate information in light of the spread of false information (van der Meer & Jin, 2020) and the requirement for innovative tactics to increase readiness (Pennycook et al., 2020) against upcoming infodemics.

A number of social and psychological factors of sharing misinformation on social media are identified in several studies. Hence, a systematic review is needed to thoroughly and comprehensively investigate these factors individually discussed in separate studies. This literature review aims to summarize all the factors without any geographical or theoretical limitations. A recent systematic review on COVID-19 infodemic by Pian et al. (2021) have labeled social media usage as a cause of the infodemic. However, it does not further identify the factors that motivate individuals to share misinformation on social media. Hence, the present study is rigorously focused on social media misinformation sharing behavior, its causes, and consequences to enhance our understanding, summarize the current research, and provide suggestions for future research. Specifically, the objectives of this systematic review are to:

- Identify the possible causes or motivators of misinformation sharing behavior about the COVID-19 on social media
- Indicate the consequences of misinformation sharing behavior during COVID-19 on social media

This study is multi-disciplinary, and its outcome can also be extended to understand misinformation sharing behavior of social media users in general.

## Methods

Systematic reviews are considered a vital tool for comprehensively summarizing evidence on a particular topic to inform policy and practice (Tranfield et al., 2003). Keeping in view the study’s objectives, the Preferred Reporting Items for Systematic Reviews and Meta-Analyses PRISMA (2020; Page et al., 2021) checklist was used for the following reasons: Firstly, it is a widely used research protocol to conduct systematic reviews in research domains related to health. Various medical leading journals such as British Medical Journal, LANCET, etc., recommend this research protocol for undertaking systematic reviews and meta-analyses. Secondly, it provides guidelines to authors on ensuring a transparent and complete reporting of each section of a systematic review. It requires authors to mention the rationale and objectives of the systematic review, the search strategy used, and eligibility criteria which enhance the robustness of the study.

### Search Strategy and Data Sources

**Figure 1.**
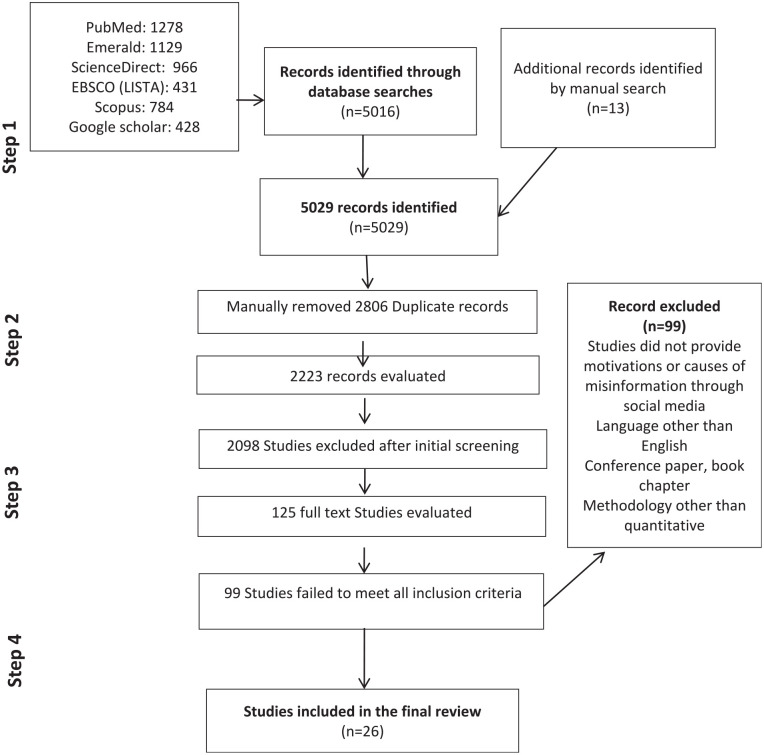
PRISMA flow diagram.

For this SLR, to undertake a concept mapping, the researchers considered misinformation as inaccurate information shared either intentionally or unintentionally on social media during COVID-19. The researchers devised a comprehensive search strategy to explore the relevant literature on five leading databases: Scopus, ScienceDirect, Emerald, EBSCO (LISTA), and PubMed using Punjab University Library portal. To avoid bias and to conduct comprehensive research, the first two researchers individually and systematically searched for relevant literature until September 30^th^ 2021. Furthermore, literature was also obtained by manual searching from core medical sciences journals, review articles, key studies using backwards and forward citation, and Google scholar. Searches were limited to title, abstract, and keywords to direct all results into the topic of COVID-19 and disinformation or misinformation without placing any restrictions on the dates or geographic location. We selected different sets of keywords for searching, such as “COVID” or “COVID-19” or “SAR-COV-2” AND “disinformation” or “misinformation” or “fake news” or “false information” or “infodemic,” along with various combinations of “factors” or “effects” or “challenges” or “causes” or “motivations” or “sources” or “predictors” or “association” or “indicators” or “reasons.” The detailed screening and selection process is shown in Figure 1 depicts the four-stage flow of the review process.

### Eligibility Criteria

The inclusion and exclusion criteria used to screen and select studies for this systematic review is given below. Those studies which me tall of the inclusion and exclusion criteria were consider for the present study.

The following inclusion criteria were followed to include an article in this review:

i. The focus of an article was on the causes or motivations and consequences of sharing mis information on social media during COVID-19 rather than it was mentioned merely as the context.
ii. Studies addressing misinformation through social media during the COVID-19 pandemic.
iii. Quantitative survey published in peer-reviewed journals.
iv. Not specific to a certain sector, such as health, tourism, or education sector.
v. Written in English language.
The following exclusion criteria were followed to exclude an article in this review:
i. Not discuss misinformation sharing through social media.
ii. Non-peer-reviewed research studies such as conference papers, reports, dissertations, textbooks, systematic reviews and book chapters.
iii. Letters, editorials, and comments.

### Study Selection and Data Extraction

A number of techniques were employed to make sure that each article was relevant and made a significant contribution to this research. To choose appropriate literature to include, the first two authors used a practical screening procedure. According to Stapleton et al. (2020)“An essential but time-consuming step in a process to find relevant evidence resources is systematic screening.”

The retrieved research studies were first examined based on their relevance and 5,029 research studies were short listed. After removing 2,806 duplicates articles, 2,223 studies were retained and evaluated. At this stage, 2,098 studies were excluded after initial screening (Figure 1). Following this, 125 full-text studies were manually arranged in a MS-Excel spreadsheet and evaluated for compliance with the purpose of this research study. This evaluation omitted 99 studies that failed to meet inclusion criteria. At the final step, 26 studies were employed to conduct this systematic analysis to reveal patterns of existing research about motivations and consequences associated with misinformation sharing behavior on social media.

## Results and Discussion

For this extensive review, we identified 26 studies published from January 2020 to September 2021. Among them, 15 studies were published in the year 2021, and the rest of 11 were published in the previous year (i.e., 2020). Country wise distribution showed that five studies were published from the USA, four from Nigeria, followed by three from Bangladesh. Two studies each from Pakistan, India, and UK while one study each from Africa, Australia, Bahrain, Ethiopia, Korea, South Korea, Spain, and Tunisia were appeared on the topic. All the selected studies were conducted with the young adult users of social media. Analysis of the selected studies (attached as Appendix A) shows the characteristics of the selected studies for this systematic literature review.

### Popular Social Media Platforms

**Figure 2.**
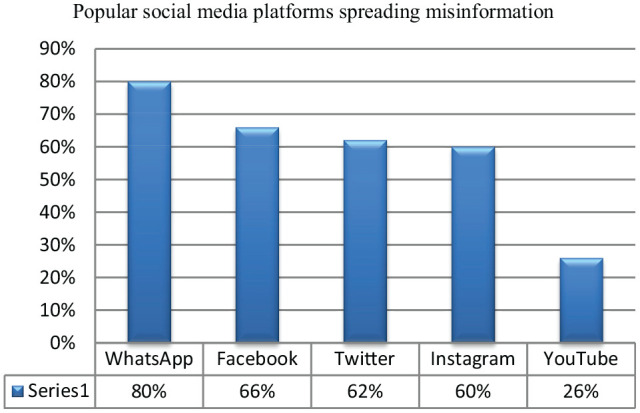
Popular social media platforms spreading misinformation.

Data analysis indicated that WhatsApp, Facebook, Twitter, Instagram, and YouTube were highly used social media platforms where misinformation related to COVID-19 was shared (Figure 2). WhatsApp was reported in 80% of the selected studies followed by Facebook (66%).

**Figure 3.**
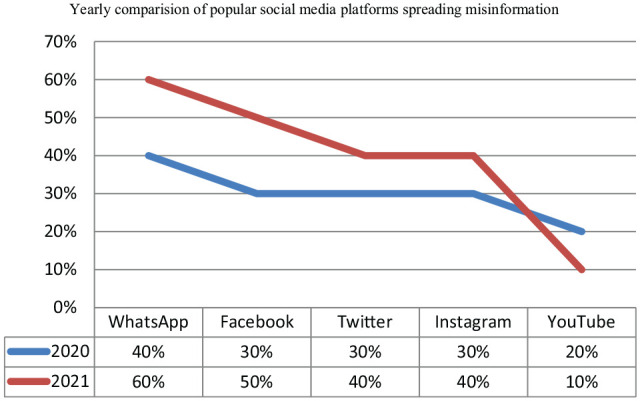
Popular social media platforms spreading misinformation-Yearly Comparison.

In comparison to the year 2020, more studies were published in the year 2021 (58%) than in the previous year (42%). Yearly comparison of the studies indicated the popularity of these social media platforms during the years 2020 and 2021 (Figure 3) was also made. The results revealed that during the both years, WhatsApp remained as the most listed platform for sharing misinformation related to COVID-19 followed by Facebook. However, more studies published in 2021 mentioned Instagram and Twitter than those published in 2020.Conversely, more studies indicated YouTube as a platform for spreading misinformation in 2020 than those published in the later year.

### Factors Associated with the Belief in Misinformation

**Figure 4.**
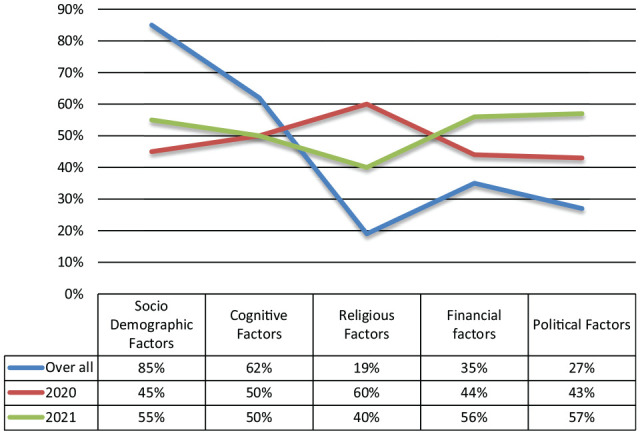
Factors associated with belief in misinformation.

Table 1 presented the factors associated with the belief in misinformation during COVID-19 as reported in the selected studies. For the comprehensive presentation, the researchers combined similar factors and discussed them under following five categories (Figure 4).

(1) Socio-demographic factors
(2) Financial factors
(3) Political factors
(4) Religious factors
(5) Conspiracy ideation

**Table 1.** Demographic Factors Associated with the Spread of Misinformation.

Factor	Variable	Source
Socio-demographicfactors	Age	Adekoya et al. (2021), Adnan et al. (2021), Agley et al. (2021), Apuke and Omar (2021), Bapaye and Bapaye (2021), Cha et al. (2021), Erinoso et al. (2021), Guelmami et al. (2021), Jamsheed (2021), Osuagwu et al. (2021), Pickles et al. (2021), Su (2021), Vijaykumar et al. (2021), Ali et al. (2020), Apuke and Omar (2020), Barua et al. (2020), Datta et al. (2020), Khalifa et al. (2020), Laato et al. (2020), Lee et al. (2020), Roozenbeek et al. (2020), Kebede et al. (2020)
	Sex	
	Education	
	Marital status	Guelmami et al. (2021), Osuagwu et al. (2021), Ali et al. (2020), Lee et al. (2020), Roozenbeek et al. (2020);
	Health status	Cha et al. (2021), Guelmami et al. (2021), Islam et al. (2020)
	Occupation	Adekoya et al. (2021), Adnan et al. (2021), Agley et al. (2021), Apuke and Omar (2021), Bapaye and Bapaye (2021), Cha et al. (2021), Erinoso et al. (2021), Guelmami et al. (2021), Jamsheed (2021), Osuagwu et al. (2021), Pickles et al. (2021), Su (2021), Vijaykumar et al. (2021), Ali et al. (2020), Apuke and Omar (2020), Barua et al. (2020), Kebede et al. (2020)
Financial factors	Financial status	Cha et al. (2021), Erinoso et al. (2021); Osuagwu et al. (2021), Su (2021), Vijaykumar et al. (2021), Ali et al. (2020), Apuke and Omar (2020), Barua et al. (2020), Kebede et al. (2020)
Political factors		Pickles et al. (2021), Ali et al. (2020), Roozenbeek et al. (2020)
	Political orientation & interest	Agley et al. (2021), Freiling et al. (2021); Su (2021), Ali et al. (2020), Lobato et al. (2020), Roozenbeek et al. (2020)
Religious factors	Cultural and Religious beliefs	Agley et al. (2021), Osuagwu et al. (2021), Ali et al. (2020), Islam et al. (2020), Kebede et al. (2020)
Conspiracy ideation	Conspiracy ideation	Islam et al. (2020), Fernández-Torres et al. (2021)
	Believe in premature &unconfirmed remedies for treatment	Bapaye and Bapaye (2021), Lobato et al. (2020)

(1) *Socio-demographic factors*: Data analysis shows that socio-demographics associated with the belief in misinformation were age (older adults), employment status (unemployed), gender (female), and education (undergraduate degree). According to Bapaye and Bapaye (2021), particular age groups are more likely to believe in and influenced by COVID-19-related misinformation. The result indicated that older people with more than 65 years of age were the most while the younger adults with less than 18 years of age were the least vulnerable to misinformation on social media. Similarly, females were more susceptible to belief in misinformation than male. Several studies indicated that unemployed and less educated people were more prone to have belief in misinformation (for reference please see; Adekoya et al., 2021; Apuke & Omar, 2020; Bapaye & Bapaye, 2021; Barua et al., 2020; Datta et al., 2020; Lee et al., 2020). Marital status also has associated with misinformation beliefs. Osuagwu et al. (2021) indicated that persons who are not married, which included single, divorced, separated, or widowed were significantly associated with the misinformation beliefs. Few studies indicated association between health status and misinformation as well (Cha et al., 2021; Guelmami et al., 2021; Islam et al., 2020). Numerous studies find the relationship of occupation and misinformation beliefs during Covid-19 (Adnan et al., 2021 & Osuagwu et al., 2021). Bapaye and Bapaye (2021) mentioned that the vulnerability of those users who were involved in elementary occupations was significantly higher than other professionals and students. The study also indicated that health care workers, who are otherwise considered as experts with regard to this global health care crisis, also shared this vulnerability to misinformation with other occupation groups.

(2) *Financial factors*: were mentioned by a number of studies. Data analysis of the selected studies shows a positive association between the financial status and disinformation sharing on social media during the COVID-19 pandemic. Numerous studies highlighted that low-income countries or person from low economic status might be at a higher risk of exposure to misinformation (Ali et al., 2020; Apuke & Omar, 2020; Barua et al., 2020; Cha et al., 2021; Erinoso et al., 2021; Su, 2021; Vijaykumar et al., 2021).

(3) *Political Factors*: Several authors pointed out that there was a strong association of political affiliation or interests with misinformation belief and sharing during the COVID-19 pandemic (Ali et al., 2020; Freiling et al., 2021; Lobato et al., 2020; Roozenbeek et al., 2020; Su, 2021). Fernández-Torres et al. (2021) indicated that premature statements and contradictory messages by political representatives of different parties as well as government bodies was one of the major reasons behind the misinformation sharing during the COVID-19.

(4) *Religious Factors*: The literature regarding religious factors was mixed of nature; some studies supporting that, religious beliefs influence misinformation sharing while others were not in favor of this hypothesis. A few studies reported that religious beliefs or commitments were also associated with the misinformation belief and sharing on social media during the COVID-19 crisis (Agley et al., 2021; Ali et al., 2020; Islam et al., 2020; Osuagwu et al., 2021; Kebede et al., 2020). For instance, conducted a study of the USA adults reported that religious leaders were among the most trusted sources for Covid-19 related information (Ali et al., 2020). Kebede et al. (2020) highlighted that people considered their religious beliefs enough to control COVID-19 virus. Conversely, Agley et al. (2021) declared that religious commitment were marginally, or non-significantly associated with misinformation sharing during COVID-19.

On the other hand, Islam et al. (2020) indicated that religiosity had positive influence on misinformation sharing through social media during the Covid-19 emergency. That means religious persons were more prone to believe in misinformation and were likely to share it through social media (Agley & Xiao, 2021; Ali et al., 2020; Osuagwu et al., 2021; Kebede et al. 2020). It is important to mention that the studies which discussed the religious aspects were mostly conducted with Muslim population. So, the findings may not be generalizable for the people of other religions.

(5) *Conspiracy Ideation*: The studies also indicated that people with conspiracy ideation were more susceptible to believe in and further share misinformation. Low level of trust in science and governments, and high belief in traditional remedies and traditionalism were the listed reasons leading to believe in conspiracy theories. Lobato et al. (2020) found that individuals with a high degree of traditionalism but low social authority were more likely to disseminate misinformation on social media during the COVID-19 crisis. He further hypothesized that persons who relied more on their intuitions and lacked basic scientific understanding were less adept at distinguishing between genuine and incorrect information (in terms of both accuracy and sharing decisions). Bapaye and Bapaye (2021) indicated an interesting paradox behind misinformation sharing during Covid-19, that people are more eager to take unverified remedies through social media but not willing to accept evidence-based advice.

Table 1 shows the studies indicated the positive association of these factors and beliefs associated with misinformation sharing behavior.

### Predictors of Misinformation Sharing

Misinformation emerged as a serious challenge that attracted researchers to explore the motivators of sharing misinformation on social media during the COVID-19 crisis. The motivating factors of sharing misinformation on social media were identified and summarized in Table 2 below:

**Table 2.** Extracted Predictors of Misinformation Sharing-Source.

Predictors of misinformation sharing	Sub-categories	Source
Entertainment	Entertainment	Adekoya et al. (2021), Adnan et al. (2021), Apuke and Omar (2021), Barua et al. (2020), Islam et al. (2020)
	Time pass	Adnan et al. (2021), Apuke and Omar (2021)
Information-seeking	Information-seeking	Adekoya et al. (2021), Adnan et al. (2021), Apuke and Omar (2021), Ali et al. (2020), Apuke and Omar (2020)
	Information-sharing	Adnan et al. (2021), Apuke and Omar (2021)
	Exploration	Islam et al. (2020)
Information overload		Laato et al. (2020), Lee et al. (2020);
Socialization	Socialization	Adnan et al. (2021), Apuke and Omar (2021, 2020), Lobato et al. (2020)
	Social tie strength	Apuke and Omar (2021), Lobato et al. (2020)
	Social Norms	Jamsheed (2021)
	Para-social interaction	Apuke and Omar (2020), Roozenbeek et al. (2020)
Status-seeking	Status-seeking	Apuke and Omar (2020), Khalifa et al. (2020)
	Self-promotion	Barua et al. (2020), Islam et al. (2020);Apuke and Omar (2020), Khalifa et al. (2020)
	Perceive to be helpful	Islam et al. (2020), Khalifa et al. (2020)
Trust	Low trust in science	Agley et al. (2021), Pickles et al. (2021); Lobato et al. (2020), Roozenbeek et al. (2020)
	Online information trust	Laato et al. (2020), Lee et al. (2020)
	Perceived accuracyof information	Su (2021), Lee et al. (2020)
Anxiety	Anxiety	Osuagwu et al. (2021), Pickles et al. (2021), Freiling et al. (2021), Guelmami et al. (2021), Su (2021), Datta et al. (2020), Roozenbeek et al. (2020), Kebede et al. (2020)
	Fear	Pickles et al. (2021), Freiling et al. (2021), Guelmami et al. (2021), Su (2021), Datta et al. (2020), Roozenbeek et al. (2020)
	Perceived susceptibility	Laato et al. (2020), Lee et al. (2020), Roozenbeek et al. (2020)
	Perceived severity	Laato et al. (2020), Lee et al. (2020)
	COVID-19 risk perception	Freiling et al. (2021), Guelmami et al. (2021); Su (2021), Datta et al. (2020), Kebede et al. (2020); Roozenbeek et al. (2020), Osuagwu et al. (2021)
Self-efficacy	Self- using social media	Jamsheed (2021), Pickles et al. (2021), Khalifa et al. (2020)
	digital health literacy	Guelmami et al. (2021), Islam et al. (2020); Jamsheed (2021), Khalifa et al. (2020), Pickles et al. (2021), Roozenbeek et al. (2020)
Altruism	Altruistic motivation & pro-social behavior	Adnan et al. (2021), Apuke and Omar (2021), Fernández-Torres et al. (2021)
Deficient self-regulation	Deficient self-regulation	Islam et al. (2020)
	Internet addiction	Guelmami et al. (2021); Apuke and Omar (2020), Islam et al. (2020)
	SNS dependency	Su (2021), Islam et al. (2020)
	SNS frequency	Adekoya et al. (2021), Bapaye and Bapaye (2021), Guelmami et al. (2021), Jamsheed (2021), Su (2021), Apuke and Omar (2020), Barua et al. (2020), Datta et al. (2020), Khalifa et al. (2020)

**Figure 5.**
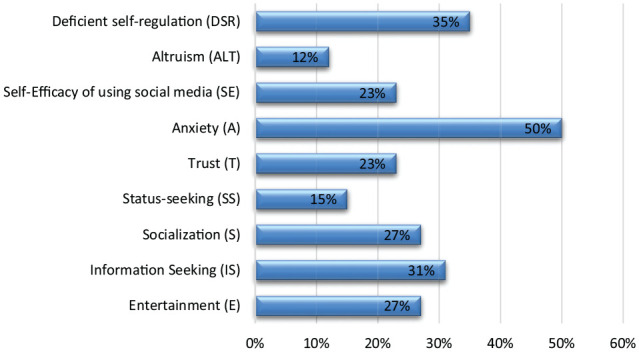
Predictors of misinformation sharing-percentage.

The results indicated that a good number of studies mentioned that anxiety, fear, or perceived risks of COVID-19 was indicated as one of the major motivators of misinformation sharing (Figure 5), followed by the entertainment, information-seeking, socialization/, self-promotion, lack of trust in science, lack of self-efficacy, and altruistic motivation respectively. The studies conducted by Guelmami et al. (2021), Osuagwu et al. (2021), Su (2021), and Roozenbeek et al. (2020) indicated a significant association between the perceived risk of getting COVID-19 and the spread of misinformation on social media.

**Figure 6.**
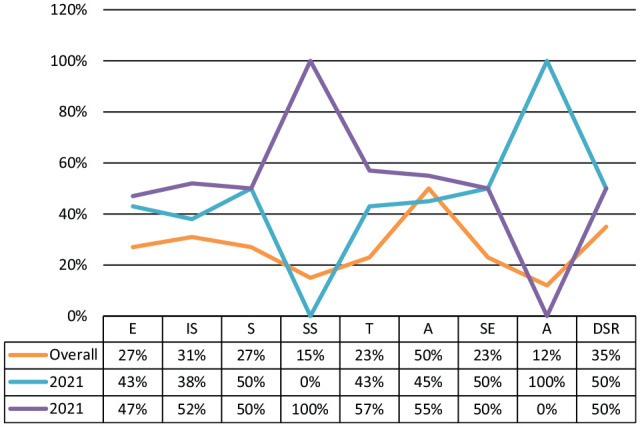
Predictors of misinformation sharing**-**yearly comparison.

Yearly comparison as shown in Figure 6 found a very interesting shift among predictors or causes of misinformation sharing on social media during 2020 and 2021. For instance, studies published in 2020 (*n* = 11), had discussed more causes of sharing misinformation during Covid-19 than those published in 2021 (*n* = 15). In addition to, the motivators such as status seeking, deficient self-regulation, online information trust and information seeking were mentioned by many studies published during 2020 but not found in the next year’s studies (Figure 6). It is pertinent to mention that. On the other side, altruistic motivation, information-seeking and socialization were less highlighted during 2020 and more in 2021.

The findings indicated that fear or risk perception associated with COVID-19 was one of the most often stated motivators for disinformation sharing via social media. Vijaykumar et al. (2021) These findings could be explained by a lack of understanding of fact-checking sites, ignorance of current misinformation, and positive reinforcement from echo chambers. Socio-demographic characteristics were the most frequently addressed factors associated with disinformation sharing and COVID-19. According to Laato et al. (2020), trust in online information and perceived information overload are significant determinants of unverified information sharing.

Apuke and Omar (2020) mentioned that social-tie strength was the strongest predictor and positively associated with sharing misinformation on social media during COVID-19 pandemic. They believe that the information obtained from a strong social-tie source is perceived as more trustworthy. Their study also verified that people were more likely to trust the information shared by their acquaintances and less bothered to verify from other sources. Studies discussing the relationship of entertainment and status- seeking with misinformation sharing behavior are of mixed results. Barua et al. (2020) and Islam et al. (2020) identified that self-promotion, entertainment, and status seeking were the major reasons of sharing unverified COVID-19 information on social media. Contrary to these studies, some studies came up with different findings. These studies believed that those people who are motivated to self-promote themselves are less likely to share fake news. Furthermore, they also indicated that entertainment had no association with fake news sharing on COVID-19 (i.e., Apuke & Omar, 2020, 2021; Laato et al., 2020; Talwar et al., 2020). However, other studies (i.e., Adekoya & Fasae, 2022; Adnan et al., 2021; Ali et al., 2020) found that information seeking and self-promotion, and entertainment as significant predictors of sharing misinformation related to COVID-19 on social media.

#### Consequences of misinformation sharing

Misinformation negatively influenced human society during the Covid-19 crisis. Nelson et al. (2020) labeled misinformation as a powerfully destructive force in this era of interactive global communication. The spread of rumors, stigma, and conspiracy theories not only affected individuals but also institutions and societies at large. WHO (2020) reported that misinformation related to COVID-19 was polarizing societies by amplifying hate speech, social stigma, gender disparities, and generational rifts.

**Figure 7.**
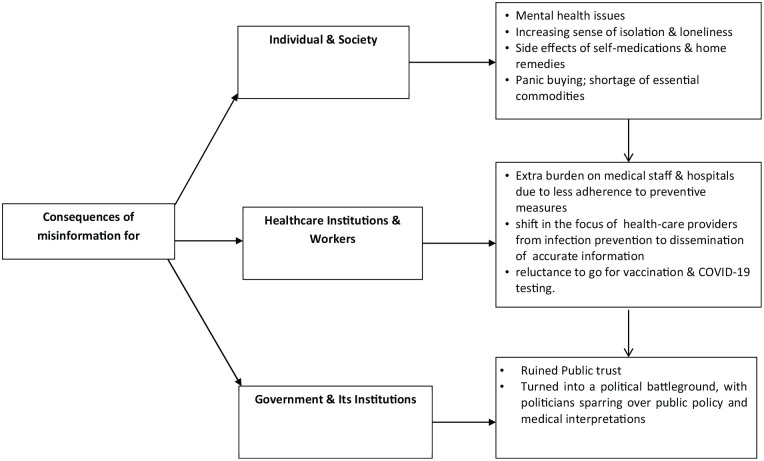
Consequences of misinformation during Covid-19.

The spread of misinformation impacted the individuals’ behavior in many different ways (Figure 7). First, it created panic among general public. For example, the news of complete lockdown spread through social media sparked panic-buying that adversely affected demand and supply chain in many countries. As a result, essential items such as face masks, hand sanitizers, and toilet paper became out of reach as a result of this panic buying (Barua et al., 2020). Secondly, locked down and other restrictions led to the increased sense of loneliness among individuals and they turned to social media to interact with the outer world (Boursier et al., 2020). The surging waves of misinformation on social media contributed to mental health issues such as worry, stress, and depression (Su, 2021). The analysis of the selected studies indicated (Figure 6) that misinformation about COVID-19 negatively affected individuals’ mental health and psychological well-being. Due to misinformation, fear and bewilderment spread across the communities which triggered people to do things that had little to do with the adherence of health protective behavior or curtailing the spread of the disease (Su, 2021; Zhao & Zhou, 2020). During the pandemic, misinformation masqueraded itself as true infection prevention and control strategy, which brought adverse consequences for individuals. Wrong precautions, self-medications, and home remedies caused fatal consequences and posed extraordinary challenges to public health systems. Review of the relevant literature reveals several documented cases where individuals were found following inappropriate and misguided strategies to prevent the disease (Ali et al., 2020; Laato et al., 2020; Osuagwu et al., 2021; Pickles et al., 2021; Roozenbeek et al., 2020). On the other hand, in certain cases, rumors, conspiracy theories and fake news led individuals to show less compliance with health protective behavior as well as low intention to get vaccination (Barua et al., 2020; Datta et al., 2020; Islam et al., 2020; Su, 2021; Vijaykumar et al., 2021).

The widespread dissemination of fake information caused a sever burden on health professionals. They had to treat not only COVID patients but also those who got harmed due to wrong self-medications and home remedies while following misinformation. This situation posed extra burden on health-care providers and health institutions to combat misinformation by sharing authentic and credible information with scientific evidences. Scholars have raised concerns about the consequences of widespread misinformation on a variety of health-related topics. Avoidance of preventative measures and the decrease of people’s awareness of the harmfulness of Covid-19 virus are two of these outcomes. (Allington et al., 2021; Naveed et al., 2021).

Trust on healthcare providers, health institutions and government was considered critical to deal with the Covid-19 pandemic. However, Banerjee and Meena (2021) portrayed that an abundance of mixed quality information led to information pollution, making it impossible to discern “what is authentic” and ultimately damaged public trust in governments, medical science, health care professionals, and institutions. Nelson et al. (2020) indicated that the pandemic has turned into a political battleground, with politicians sparring over public policy and medical interpretations that resultantly created more confusion about the disease and lesser trust on governments, science, and politicians.

#### Theoretical implications

Our study contributes to the literature on misinformation sharing behavior in several ways. First, prior literature discusses the scarcity of empirical studies investigating comprehensively into the psychological motivators influencing misinformation sharing behavior on social media, which was not thoroughly investigated by previous literature (Bin Naeem & Kamel Boulos, 2021; De Coninck et al., 2021; Skafle et al., 2022; Talwar et al., 2020). This study summarized those factors without any geographical or theoretical limitations, and presented them collectively while grouping them into different conceptual categories. Therefore, our research has provided comprehensive dimension to theoretical underpinnings of the phenomenon at hand and assembled the consequences of infodemic discussed by the selected studies. we collected and summarized the influential factors particularly in the context of Covid-19 pandemic; hence the contribution may be limited to this particular event. Though, further investigation is required to extend the generalization of this study findings, still they are helpful to enhanced our overall understanding regarding unverified information sharing behavior on social media during any crisis., Overall, this study is multi-disciplinary, and its outcome can also be extended to understand misinformation sharing behavior of social media users in general. The results of our study are generalizable due to the diversity of population from different cultural contexts along with the variety of theoretical perspectives discussed in the selected studies.

Secondly, our study identified and separated the positive factors from the negative ones. For instance, socialization, anxiety, trust, altruism, information overload, and deficient self-regulation (DSR) positively, while information-seeking, and self-efficacy negatively influenced misinformation sharing behavior of individuals during Covid-19 pandemic. This implies that these two constructs can be thought of as coping strategies which help individuals to refrain from sharing unverified information on social media. Studies suggested that individuals, competent in searching online information, and using social media could confidently assess the quality of shared information and combat misinformation on social media (Apuke & Omar, 2020). Several studies also suggested that digital health literacy would be crucial to curtail misinformation sharing on social media (Adekoya & Fasae, 2022; Erinoso et al., 2021; Lee et al., 2020; Pickles et al., 2021). Furthermore, we found that entertainment and status seeking were contextual and behaved differently in different cases and scenarios. In certain studies, both of the constructs positively while in other negatively predicted the misinformation sharing behavior of social media users. This implies that depending on different situations and cases, entertainment and status seeking may lead to both positive and negative effect on the behavior of sharing misinformation.

#### Practical implications

Besides these theoretical implications, this study also offers practical implications while providing a comprehensive explanation of all the factors which positively or negatively influence the misinformation sharing behavior of social media users. Such understanding will pave the way for all stakeholders (i.e., behavioral scientists, public health researchers, and policy makers) to take steps to alleviate their effects in society. The negative influence of information seeking self-efficacy, digital health literacy elucidates the significance and need of training people in identifying, seeking, and searching information from authentic and credible online information sources. It is essential to educate and train social media users to recognize and refute misinformation on social media by designing digital information literacy programs. Even though certain social media platforms have taken steps to limit the transmission of COVID-19-related misinformation by eliminating fact-checked erroneous and potentially dangerous content, still it is challenging to curtail misinformation on social media. Barua et al. (2020) suggested that social media users should be encouraged and trained to assess and evaluate the quality of shared information by rechecking it with trusted sources before taking any further decisions. The findings also provide insights to social media platform developers/designers to design such content which can be double-checked and shared easily

Socio-cultural change in handling information on social media is also suggested. For example, the results show positive dual relationship (both positive and negative) of self-promotion and entertainment with misinformation sharing behavior. It indicates the need of making individuals realized that misinformation sharing can destroy their positive image. Islam et al. (2020) think that social media users deleted their posts immediately when they identified that their shared information is fake. Moreover, it is essential to make people realized that sharing misinformation and fake news merely out of fun can be disastrous during crisis. Individuals, institutes and governments can run awareness campaigns on how to cope with infodemic and why it is crucial to show responsible sharing behavior on social media.

The result of this study articulates that information overload and anxiety (i.e., fear or risk perception) associated with COVID-19 were one of the significant motivators for misinformation sharing (Barua et al., 2020; Datta et al., 2020; Freiling et al., 2021; Guelmami et al., 2021; Osuagwu et al., 2021; Su, 2021; Kebede et al. 2020). Social media has made dissemination of information easy, speedy and widespread which contributing to information overload. Here come the role of institutions and governments to ensure that people receive timely, accurate, and manageable amounts of updates which will be easily understandable and consumable (Chen et al., 2021; Kulkarni & Anantharama, 2020). Furthermore, findings also suggested that while crafting, selecting and promoting information related to COVID-19, positive and optimistic content would better serve the purpose. Furthermore, the results of this review emphasize the need of considering individual characteristics such as age, financial status, religion, and political affiliation while creating and sharing content on social media. It is suggested to employ users’ segmentation strategy and offer customized content and training for social media uses to combat misinformation during any crisis.

#### Limitations and future research

The study has some limitations as well. First, while the search was confined to papers published in English, there may be valuable studies published in other languages that were not included in this study. Second, despite using five abstracting databases and scanning a vast number of papers to maximize the search, some significant research may have been overlooked. As this SLR focuses solely on quantitative studies, hence, studies are overlooked from a qualitative standpoint which might be a topic of interest for future research. Small sample size (*n* = 26) could be considered as a limitation as well.

Furthermore, this study only looked at the predictors of misinformation sharing behavior during COVID-19. It did not look into the factors that could curb the sharing of misinformation on social media platforms. Future SLR studies may be conducted to investigate the factors that combat or curtail the spread of misinformation.

#### Concluding comments

COVID-19 misinformation is pervasive and has been fast spreading across the globe via social media channels. Widespread misinformation was a major concern to public health during the COVID-19 pandemic. This study contributes to existing knowledge by identifying the reasons and the factors influencing misinformation sharing behavior on social media platforms.

Such understanding of the underlying reasons for misinformation sharing will ensure improved public health communications, and effective efforts to prevent compromised viral transmission.

Lee et al. (2020) discovered a strong association between disinformation exposure and younger age, greater education levels, and lower income and proficiency in digital health.

## Appendix

**Appendix A.** Characteristics of Selected Studies.

Author	Journal	Country	Population	Statistical Test
Khalifa et al. (2020)	Arab Media & Society	Bahrain	1,274 Adults from middle east countries	Descriptive statistics
Lee et al. (2020)	Journal of Medical Internet Research	South Korea	1,049 South Korean adults	Descriptive statistics
Ali et al. (2020)	JMIR Public Health and Surveillance	USA	11,242 US adults	Multivariable logistic regression analyses
Apuke and Omar (2020)	Health Education Research	Nigeria	650 age 18+ social media users in Nigeria	Structural equation model with SmartPLS
Barua et al. (2020)	Progress in Disaster Science	Bangladesh	483 Bangladesh respondents	Partial Least Square (PLS)
Datta et al. (2020)	Medical Journal Armed Forces India	India	758 Medical students	Chi-square test
Islam et al. (2020)	Technological Forecasting & Social Change	Bangladesh	433 Young adults in Bangladesh	PLS-SEM
Laato et al. (2020)	European Journal of Information Systems	Bangladesh	294 Students, faculty and alumni of two universities in Bangladesh	PLS-SEM
Lobato et al. (2020)	Frontiers of Psychology	USA	296 US adults	Descriptive statistics
Roozenbeek et al. (2020)	Open Science	UK	5,000 Adults from the UK, Ireland, USA, Spain & Mexico	OLS & linear regression one-way analysis of variance
Kebede et al. (2020)	PLoS ONE	Ethiopia	929 Ethiopian adults	One-way ANOVA and t-test, standard deviations
Osuagwu et al. (2021)	Health Security	African Countries	1969 Adults 18 years or older from African countries	Multinomial logistic regression
Adekoya et al. (2021)	Global Knowledge, Memory and Communication	Nigeria	1,200 Social media users above 18 years	Descriptive & inferential statistics
Adnan et al. (2021)	Research Journal of South Asian Studies	Pakistan	385 Adults having an active account on any social media platform	Descriptive statistics& linear regression analysis
Agley et al. (2021)	Agley and Xiao BMC Public Health	USA	660 US-based Amazon Mechanical Turk (mTurk) users	Descriptive statistics & multivariate regression models
Apuke and Omar (2021)	Telematics and Informatics	Nigeria	385 Nigerian adults18 and above	Partial Least Squares (PLS)
Bapaye and Bapaye (2021)	JMIR Public Health and Surveillance	India	1,137 Whatsapp users in India	Kmean; g 2-tailed z-test.
Cha et al. (2021)	JMIR Human Factors	Korea	1,257 Social media users from 35 countries	Descriptive statistics
Erinoso et al. (2021)	JMIR Public Health and Surveillance	Nigeria	719 Adults residing in Nigeria	Descriptive statistics
Fernández-Torres et al. (2021)	Int. J. Environmental Research & Public Health	Spain	1,115 Spanish social media users	Descriptive statistics
Freiling et al. (2021)	New Media & Society	USA	719 US citizen ages ranged from 18 to 76	Linear regression analysis
Guelmami et al. (2021)	JMIR Formative Research	Tunisia	874 Tunisian adults	Descriptive statistics & Pearson correlation tests
Jamsheed (2021)	Library Philosophy and Practice	Pakistan	408 Law student’s from Pakistan	Descriptive statistics
Pickles et al. (2021)	Journal of Medical Internet Research	Australia	9,619 Australian 18+	Descriptive statistics
Su (2021)	Telematics and Informatics	USA	482 American citizens of voting age	T-Test, bivariate correlations & Hierarchical multiple regression
Vijaykumar et al. (2021)	Humanities and Social Sciences Communications	UK	1,454 UK and Brazil adult Whatsapp users	Descriptive statistics & t-tests
